# Footprints reveal direct evidence of group behavior and locomotion in *Homo erectus*

**DOI:** 10.1038/srep28766

**Published:** 2016-07-12

**Authors:** Kevin G. Hatala, Neil T. Roach, Kelly R. Ostrofsky, Roshna E. Wunderlich, Heather L. Dingwall, Brian A. Villmoare, David J. Green, John W. K. Harris, David R. Braun, Brian G. Richmond

**Affiliations:** 1 Department of Human Evolution, Max Planck Institute for Evolutionary Anthropology, Deutscher Platz 6, D-04103 Leipzig, Germany; 2 Center for the Advanced Study of Human Paleobiology, Department of Anthropology, The George Washington University, 800 22^nd^ St., NW, Suite 6000, Washington, DC 20052, USA; 3 Department of Human Evolutionary Biology, Harvard University, 11 Divinity Ave., Cambridge, MA 02138, USA; 4 Division of Anthropology, American Museum of Natural History, New York, NY 10024, USA; 5 Department of Biology, James Madison University, MSC 7801, Harrisonburg, VA 22807, USA; 6 Department of Anthropology, University of Nevada Las Vegas, Las Vegas, NV 89154, USA; 7 Department of Anatomy, Midwestern University, 555 31^st^ St., Downers Grove, IL 60515, USA; 8 Department of Anthropology, Rutgers University, New Brunswick, NJ 08901, USA

## Abstract

Bipedalism is a defining feature of the human lineage. Despite evidence that walking on two feet dates back 6–7 Ma, reconstructing hominin gait evolution is complicated by a sparse fossil record and challenges in inferring biomechanical patterns from isolated and fragmentary bones. Similarly, patterns of social behavior that distinguish modern humans from other living primates likely played significant roles in our evolution, but it is exceedingly difficult to understand the social behaviors of fossil hominins directly from fossil data. Footprints preserve direct records of gait biomechanics and behavior but they have been rare in the early human fossil record. Here we present analyses of an unprecedented discovery of 1.5-million-year-old footprint assemblages, produced by 20+ *Homo erectus* individuals. These footprints provide the oldest direct evidence for modern human-like weight transfer and confirm the presence of an energy-saving longitudinally arched foot in *H. erectus*. Further, print size analyses suggest that these *H. erectus* individuals lived and moved in cooperative multi-male groups, offering direct evidence consistent with human-like social behaviors in *H. erectus*.

Bipedal locomotion was a key adaptation of the human lineage, enabling our ancestors to travel efficiently on the ground^1^ and freeing the hands for the adoption of other uniquely human behaviors. Although the earliest hominins were likely habitual bipeds^2^,^3^,^4^, how and in what contexts different forms of bipedalism evolved, and when a fully human-like gait first emerged are subjects of considerable debate. The retention of climbing-related traits alongside bipedal adaptations in members of the genus *Australopithecus*^5^ and the earliest members of *Homo*^6^ have led to conflicting interpretations regarding the extent to which these hominins’ bipedal gaits were similar to the gait of modern humans^7^,^8^. In comparison, it is widely assumed that the human-like overall body plan of *Homo erectus* reflects adaptations for essentially human-like bipedalism^8^,^9^.

However, there is limited fossil evidence that can be used to directly address the bipedal biomechanics of *H. erectus*, and the small samples of known evidence all suggest the possible retention of primitive postcranial traits that could significantly affect locomotion. A pelvis from Gona, Ethiopia, attributed by its discoverers to *H. erectus* (but see ref. 10), is human-like in some respects but its wide breadth and laterally flaring iliac blades distinguish this specimen and suggest locomotor differences from modern humans^11^. An assemblage of 11 isolated foot bones from Dmanisi, Georgia, which could represent the extent of our knowledge of *H. erectus* feet (although their taxonomic place within the genus *Homo* is also unclear^12^), bears some morphological similarities to the foot bones of modern humans but also exhibits morphological differences that could have important biomechanical consequences^13^. Preliminary analyses of a small sample of 1.5 Ma possible *H. erectus* footprints from Ileret, Kenya suggested an essentially human-like gait, but those authors also noted potential biomechanical differences related to the abduction angle of the hallux and the extent of medial weight transfer^14^. Those preliminary analyses compared the first set of excavated Ileret footprints to a small number of available modern human and fossil hominin tracks, and at that point it was largely unknown how and to what extent specific biomechanical patterns are preserved in footprint shapes. The limitations of these studies, as well as a dearth of relevant fossil evidence, have led to only equivocal direct support for the hypothesis that *H. erectus* bipedalism was fundamentally human-like.

Certain social behaviors such as patterns of cooperation^15^ and the sexual division of foraging behavior^16^ distinguish humans from other extant primates and likely played influential roles in human evolution. However, as is the case with locomotion, these aspects of our biology and behavior are difficult to reconstruct in fossil hominins because they are not directly preserved in skeletal fossils or archaeological materials. Indirect approaches, such as observational studies of behavior in modern hunter gatherers^15^,^16^,^17^ or predictions of group size based on predicted neocortex size^18^, have allowed researchers to develop hypotheses regarding fossil hominin group composition and group behavior. However, fossil data that would allow one to directly test these hypotheses have been lacking.

In other areas of paleontology, footprints have provided key insights through their direct records of both gait biomechanics^19^ and social behavior^20^. But in the human fossil record, footprint sites are rare and typically not of the scale necessary to address these questions. Here, we present analyses of a *H. erectus* trace fossil discovery of unprecedented scale, including 97 footprints made by at least 20 different individuals at multiple sites dated to *c.*1.5 Ma near Ileret, Kenya (Fig. 1). With these data, we assess body size and taxonomic attribution, and use human experimental data to directly test whether the fossil tracks record evidence of modern human-like locomotor patterns. Furthermore, the presence of multiple distinct trackways moving across the same footprint surfaces provides a new means to analyze and interpret the first direct fossil evidence of social group composition and behavior in *H. erectus*.

## Results and Discussion

**Figure 2:**
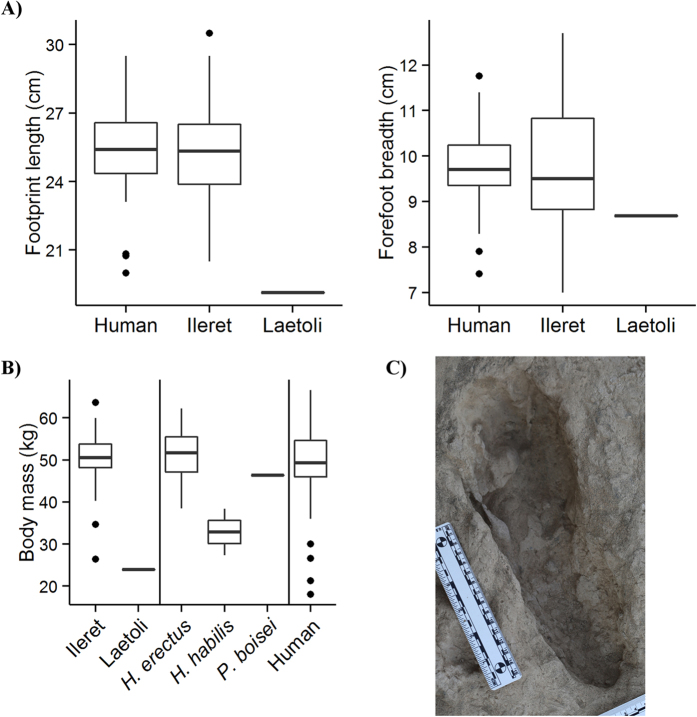
Comparisons of external footprint dimensions and body mass predictions from fossil hominin footprints. In all boxplots, the box encloses the 25–75% interquartile range, the bold line represents the median, and the upper and lower whiskers extend to the largest and smallest observations within a distance of 1.5 times the interquartile range above and below the limits of the box. (**A**) The 1.5 Ma Ileret hominin footprints are comparable in size (length and breadth) to the prints of habitually barefoot modern humans. The 3.7 Ma prints from Laetoli are considerably shorter in length and only somewhat narrower. In the figure, data are averaged by trackway (fossil tracks) or subject (human tracks). Total sample sizes are—human (n = 41 subjects, 490 footprints), Ileret (lengths: n = 28 trackways, 46 footprints; breadth: n = 36 trackways, 68 footprints), Laetoli (n = 1 trackway, 5 footprints). (**B**) Predictions of body mass from fossil track dimensions (left), are compared with body masses estimated from postcranial skeletal material of hominin species living near Ileret around 1.5 Ma (center) and measured body masses of the habitually barefoot modern human experimental sample (right). The Ileret hominin prints (n = 23 trackways) suggest much larger body masses than the prints from Laetoli (n = 1 trackway). They are more comparable in predicted mass to modern humans (n = 41 subjects) and skeletally derived estimates for *H. erectus* (n = 4) than they are to estimates for fossils attributed to *P. boisei* (n = 1) or *H. habilis* (n = 2), consistent with preliminary analyses^44^. See Supplementary Table 3 for details on fossil skeletal sample. (**C**) Photograph of an exceptionally large (>30 cm long) Ileret track, estimated to be a 58.8 kg male. Scale at left is 15 cm.

Estimates of hominin body size are typically made from a handful of measurements taken from just a few individuals separated in space and time. Calculations of body mass derived from the Ileret track assemblage offer a rare opportunity to examine this important measure at the population scale from individuals living in close proximity to each other. The Ileret footprints are generally comparable in size (length and breadth) to those of footprints produced in the same substrate by habitually barefoot Daasanach people living near Ileret today and differ markedly from the earlier 3.7 Ma Laetoli hominin tracks (Fig. 2A, Supplementary Table 1). Using experimental footprint and anthropometric data, a machine learning algorithm was built that accurately predicts an individual’s body mass from their footprint’s external dimensions (more accurately than past linear regression techniques–see Methods). Body mass estimates were calculated for well-defined footprints from 23 Ileret trackways, where the whole foot outline was visible and not distorted by depositional/taphonomic factors. Based on stratigraphic positions, depositional contexts and quantifications of footprint sizes and morphologies, we estimate that these 23 Ileret trackways from which we could estimate body mass were produced by as many as 23 or as few as 15 unique individuals (in some cases we can neither exclude nor confirm the possibility that the same individual may have produced multiple trackways within the same site). For now, we present here 23 distinct predictions. The mean predicted mass from these 23 Ileret hominin trackways was 48.9 kg (standard deviation: 9.6 kg), which is generally comparable to the body sizes of adult Daasanach individuals (mean = 52.6 kg, standard deviation = 5.9 kg for a sample of 29 adults including 15 males and 14 females). In some cases, estimates derived from the fossil tracks are on the largest end of the Daasanach range (Fig. 2B,C, Supplementary Table 2). It is also important to note that these predicted body masses from the Ileret tracks are similar to the estimates of 48–52 kg that were derived from presumed *H. antecessor* footprints at the European site of Happisburgh^21^. One outlying Ileret track implies a small body mass, and could represent a child. The length of this track, measured from the end of the heel to the tip of the hallux, is 20.5 cm and this length, according to the data collected by Ashton and colleagues^21^, is roughly equivalent to the foot size of a 9-year-old modern human.

Estimates of body size derived from the Ileret tracks support their attribution to *H. erectus*. In Fig. 2B, body mass predictions are compared with recently published estimates based on skeletal material that is most reliably attributable to the three hominin taxa known to have lived in East Africa close to 1.5 Ma^22^ (Supplementary Table 3). The majority of the Ileret tracks produced body mass estimates that fall comfortably within the interquartile range of skeletally-based estimates for *H. erectus*. All but two outliers exceeded estimates derived from *H. habilis* specimens, and the majority of mass estimates from the Ileret tracks are larger than that of the only confidently attributed *P. boisei* specimen, which has been described as an extremely robust male individual^23^. Small sample sizes of confidently attributed *H. habilis* and *P. boisei* postcrania lead to unavoidable complications in these comparisons, as does the total lack of postcrania that can be confidently attributed to *Homo rudolfensis*, a species also known from the Ileret area but with a poorly known temporal range. It remains possible that any of these sites could preserve the prints of more than one taxon but, because each of the five excavated footprint sites contains at least one set of prints with a predicted body mass that exceeds all estimates of either *P. boisei* or *H. habilis* (and falls squarely within the *H. erectus* range), this would require a level of sympatry and habitat overlap that included tolerance of multiple species traversing the same *c.*1–20 m^2^ areas within a short time span. Multiple lines of evidence regarding the anatomy, diet, and reconstructed paleoenvironments of these taxa would also complicate a hypothesis of extreme habitat overlap (Supplementary Note 1). While the presence of multiple species cannot be ruled out, it is most parsimonious at this time to infer that *H. erectus* individuals are responsible for most, if not all, of the Ileret hominin prints.

Experimental data collected from footprints of habitually unshod modern humans shows that three-dimensional track shapes preserve direct evidence of lower limb motion patterns^24^. Using only the best-preserved samples, we compared the morphologies of the Ileret footprints (n = 11 footprints from 8 trackways) and the *c.*3.7 Ma Laetoli footprints (n = 5 footprints from 1 trackway) to a large sample of habitually barefoot modern human tracks (n = 490 footprints from 41 individuals). Six of the eight Ileret hominin trackways have morphologies that are statistically indistinguishable from those of modern humans, whereas a morphology similar to that of the Laetoli footprints was never observed in the entire human sample (Supplementary Table 4). Due to the ambiguities of other fossil evidence described above, these similarities between the Ileret and modern human footprints support the initial analyses^14^ of the Ileret prints and provide the first direct fossil evidence of a human-like pattern of external foot motion in *H. erectus*, and a bipedal gait that mirrored what is seen in humans today.

**Figure 3:**
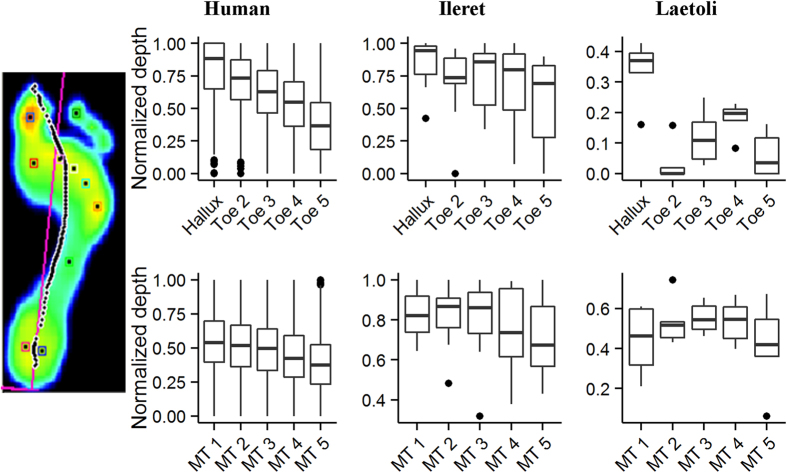
Forefoot depth profiles of modern human and fossil hominin footprints. Boxplots compare regional depth profiles of modern human footprints (n = 490 footprints from 41 individuals) to those of the 1.5 Ma Ileret (n = 11 footprints from 8 trackways) and 3.7 Ma Laetoli (n = 5 footprints from 1 trackway) hominin tracks. Top row represents depths across the toes while bottom row represents depths across the metatarsal heads. In each plot, medial is left and lateral is right. The image at far left shows the distribution of pressure including the path of the center of pressure, plotted as a dashed black line, during a typical human walking step. The overall forefoot morphology of the Ileret tracks closely resembles that of human tracks and provides evidence of a human-like medial weight transfer. The Laetoli tracks are distinct from those of humans and the Ileret hominins, and reflect a different pattern of foot biomechanics. Note that scales differ only for the purpose of better visualizing the variation within the relatively smaller fossil samples. In all boxplots, the box encloses the 25–75% interquartile range, the bold line represents the median, and the upper and lower whiskers extend to the largest and smallest observations within a distance of 1.5 times the interquartile range above and below the limits of the box.

During human walking, forces are applied to the ground in a diagnostic pattern in which greater forces are concentrated beneath the medial forefoot while the foot acts as a rigid lever with a toe-off through the first and second digits at the end of each step^25^. This medial transfer of pressure differs from the pattern of foot function observed in non-human primates, and has been consistently recognized as a defining characteristic of human foot function^26^,^27^. Modern human footprints show an overall medial-to-lateral gradient of decreasing depth across the forefoot region (metatarsal heads and toes) (Fig. 3). This reflects our unique pattern of external foot function, including a medial transfer of pressure and toe-off through the hallux and second toe. The Ileret footprints show a pattern that is slightly different but still generally similar. They may lack a clear medial-to-lateral gradient but they still show deeper impressions beneath the medial parts of the forefoot. This appears to demonstrate that a generally human-like pattern of foot function dates back to at least *H. erectus* (Supplementary Note 2). In comparison, the Laetoli prints lack this medial-to-lateral depth gradient and imply a pattern of foot mechanics and a bipedal gait that differed significantly from that of modern humans and the Ileret hominins (Supplementary Note 3). Evidence for this weight transfer pattern in the Ileret tracks, combined with shallower print depths in the medial midfoot, strongly supports the presence of a modern human-like longitudinally arched foot in *H. erectus*, which would allow considerable energy savings during long-distance walking and running^28^. The Ileret footprints thus provide new direct evidence for human-like foot anatomy and foot function in *H. erectus*, and support hypotheses of human-like functional patterns derived from the small sample of isolated foot fossils possibly attributable to this species^13^.

Multiple lines of geological, sedimentary, and taphonomic evidence suggest that the hominin tracks on any of the Ileret footprint surfaces were formed and buried within the same day, perhaps within a few hours^29^. First, many of the Ileret fossil tracks and trackways show similarly fine-detailed preservation states, and track surfaces lack evidence of soil development or root traces. The depositional context and the lack of mud cracks on any of the footprint layers suggest that these sites were rapidly buried by fine or silty sand before any drying occurred^29^. Further, modern taphonomic experiments conducted on the shores of Lake Turkana have demonstrated that human footprints retain the level of anatomical detail preserved in the fossil tracks for a maximum of 1.3 days, on average^29^. In addition to this evidence for rapid formation and burial, some of the Ileret trackways show parallel directional movement that contrasts from the directions of travel for other animals^29^. This suggests that movement was not constrained and guided by natural land features, and that hominins elected to move in the same direction. While we can never know with certainty the precise events that transpired 1.5 million years ago, these multiple lines of evidence are consistent with the hypothesis that within any one site, parallel hominin trackways could represent a group that traveled together, or at the very least a collection of individuals who co-existed and moved across the same landscape within the same day or so. The data therefore offer a rare opportunity to examine and test hypotheses about hominin group composition, which cannot be assessed directly from any other form of fossil data.

**Figure 4:**
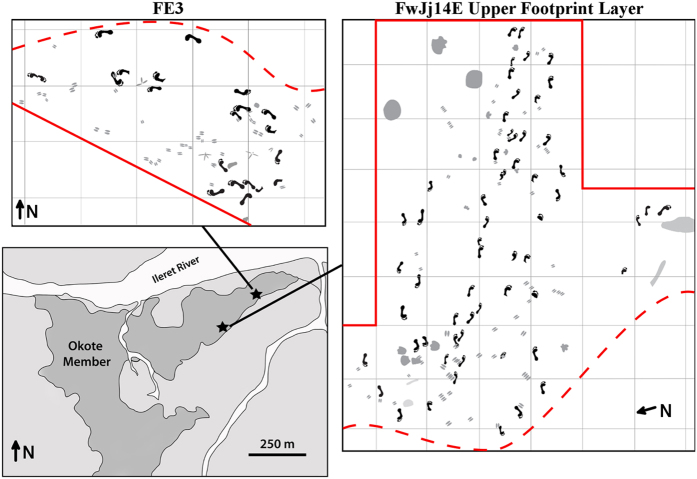
Schematic maps of excavated footprint surfaces at sites FE3 and FwJj14E. Map of the Ileret area (lower left) shows the locations of sites FE3 and FwJj14E, marked by black stars. Schematic maps of the excavated surfaces at FE3 (top left) and the FwJj14E Upper Footprint Layer (right) show the presence of multiple trackways across each of these surfaces. Print size analyses indicate that the groups of individuals represented at each site consist of predominantly males. Multiple trackways at FwJj14E show parallel directional movement and similar preservation states, suggesting that they could represent a group traveling together. Note that the schematic map of the FwJj14E surface has been rotated relative to North for visualization purposes. Solid red lines mark borders of the current excavations, and the same geological layers that preserve footprints are known to extend beyond these borders. Dashed red lines indicate the finite edge of the preserved surface, as areas beyond these lines have been lost due to erosion. The schematic map of the Ileret area was created by N.T.R., using a map generated in ArcGIS software version 10.2 (https://www.arcgis.com).

In the two most spatially-expansive footprint surface excavations, ET-2013-1A-FE3 and the Upper Footprint Layer at site FwJj14E, the presence of multiple trackways (and individuals) is clearly evident (Fig. 4). Using the mean method^30^ on our body mass predictions generated from well-preserved fossil tracks (see above), presumed sex was attributed for each of the different individuals who walked across these surfaces (Supplementary Table 2). These sex predictions indicate that a large proportion of both assemblages were likely created by adult males (3 of 4 individuals at ET-2013-1A-FE3 and perhaps as many as 8 of 16 individuals on the FwJj14E Upper Footprint Layer). This approach could conservatively underestimate the number of males present, since certain fossil discoveries^11^,^31^,^32^ and morphological analyses^22^,^33^ have evidenced that body size dimorphism in *H. erectus* was considerably higher than is observed in modern humans. If this were the case, and the size differences between male *H. erectus* and female *H. erectus* were greater than those between modern human males and females, then our use of the mean method could lead to smaller males being incorrectly classified as females^30^ (except in the seemingly unlikely scenario that the Ileret footprints were all created by female *H. erectus*, in which case male *H. erectus* body masses must have been much larger than the body masses of male modern humans and the masses that have been predicted based on *H. erectus* skeletal evidence).

It is important to note here that preservation bias can affect observed size variation in track assemblages, in that only individuals within a particular range of body masses leave discernible tracks on a substrate of a particular strength (i.e., the ‘Goldilocks’ effect)^34^. The morphologies of various tracks and trackways across a site can also be affected in disparate ways due to substrate variability across the same footprint surface^35^, such that size variation is affected in complex ways by substrate behavior. These factors may affect the diversity of sizes observed in the Ileret track assemblages, but this does not change the fact that we observe several sets of hominin footprints consistent with large overall body sizes. Regardless of the exact composition, the group of *H. erectus* individuals who at least lived in close proximity and possibly traveled together across the FwJj14E Upper Footprint Layer appears most likely to have included multiple males.

The observation of multiple *H. erectus* males interacting in close physical and temporal proximity, and possibly even moving together, on these footprint surfaces provides the first direct evidence of hominin social group composition in deep time. Among primates, cooperative male-male alliances tend to form in situations where they can provide direct advantages for accessing mates or acquiring food resources^36^. Because male-male alliances are observed in both modern humans and several modern nonhuman primates, the presence of this behavior itself in fossil hominins may be expected and unsurprising. However, the Ileret footprint surfaces offer the first opportunity to directly observe such behavior in the human fossil record.

In modern human hunter-gatherers, male-male cooperation is a key component of foraging particularly when hunting animals and subsequently sharing highly-valued meat resources^15^. Furthermore, foraging for large mammals is a high-risk strategy that may not be possible without some degree of provisioning by other individuals, often females, who typically pursue more predictably obtained foods^16^. The orientations of the hominin trackways compared to those of other animals suggests that the other animals moved to and from the water shore while hominins moved along it and, based on observations of human and other animal behavior along the modern shore of Lake Turkana, this scenario suggests the possibility that the hominins may have been foraging. The sexual division of foraging behavior is known to distinguish modern human hunter-gatherer groups from great apes and all other mammalian social carnivores^17^. If *H. erectus* was characterized by relatively high levels of sexual dimorphism^11^,^22^,^31^,^32^,^33^, and the group of *H. erectus* individuals who possibly traveled together across the FwJj14E Upper Footprint Layer consisted of all or mostly adult males (i.e., the actual number of males is greater than our estimate because we applied the mean method to a sex-imbalanced sample of *H. erectus* footprints), then these data could be evident of sexually divided foraging behavior in *H. erectus*.

We do not exclude other possibilities, however, for the types of behavior that may be represented on these footprint surfaces. For example, chimpanzees are known to form predominantly male groups during border patrols^37^. Similarly directed movement by a (possibly) predominantly male group on the Ileret footprint surfaces could reflect some type of ‘patrol’ behavior that is not unique to modern humans but instead reflects patterns of behavior with much deeper roots in our evolutionary history. The snapshots of fossil hominin behavior that are preserved by the Ileret footprint surfaces can provoke a number of alternative interpretations, and further work will be necessary in order to evaluate competing hypotheses.

Regardless of the exact behavior that was taking place, the data from multiple sites clearly show that groups of *H. erectus* individuals including multiple adult males walked together on the same landscape. These data are at the very least consistent with hypotheses that *H. erectus* had a group composition and dynamic that could have supported the emergence of human-like social behaviors such as patterns of increased cooperation and sexually-divided foraging behavior. Taken together, the data recorded within the Ileret footprint assemblages offer provocative evidence that is consistent with hypothesized grade-level shifts in the anatomy, locomotion, and behavior of *H. erectus* compared to earlier hominin taxa^8^.

## Methods

### Digital documentation of fossil hominin footprints

From 2006–2014, we excavated 24 unique sites within the *c.*1.5 Ma Ileret Tuff Complex that contained footprint surfaces and did so using a variety of survey and excavation methods. All hominin footprints that were discovered in these excavations (97 footprints from five different sites) were catalogued, mapped using a total station, measured directly (for external linear measurements such as length and breadth), and photographed in such a way that high-resolution, scaled 3-D models of each footprint could be rendered using photogrammetry software (Agisoft PhotoScan Professional, Agisoft, LLC, St. Petersburg, Russia). The same methods were used to render high-resolution 3-D photogrammetric models of first-generation casts of the Laetoli hominin footprints at the National Museums of Kenya.

### Estimates of body size derived from fossil footprints

Body size estimates were derived using a machine learning algorithm that predicted body mass using two measurements of print length (heel-to-hallux and heel-to-2^nd^ toe), two measurements of print breadth (forefoot breadth and heel breadth) and average depth across the footprint. A random forests^38^ model was built using human experimental data from a previously-published study^24^. The experimental data set consisted of measurements from 490 footprints produced by 41 Daasanach individuals, including 14 adult females, 15 adult males, 2 juvenile females, and 10 juvenile males. Across this entire sample, the average body mass was 48.6 kg (range = 18–66.6 kg, standard deviation = 10.7 kg).

To allow for robust out-of-sample tests of prediction error, human experimental data were randomly partitioned into training and test data sets comprised of 70 and 30% of the total data, respectively (n = 343 training observations and n = 147 test observations). Models were built exclusively using the training data, and later evaluated on the test data. In sum, the random forests method involves constructing a predictive algorithm that relies upon an ensemble of regression trees (in this case 500 of them) to predict body mass from the set of input variables. A complete discussion of the method has been published elsewhere^38^. Prior to the construction of each individual regression tree within the ensemble model, the training data was randomly sampled with replacement, with 217 data points used to build the model and 126 points used as the ‘out-of-bag’ sample to iteratively evaluate the model’s predictive performance.

When predictive performance was evaluated on the test observations, this random forests approach proved to generate more accurate predictions of body mass from footprint dimensions than ‘traditional’ methods that employ single linear regressions. For example, the out-of-sample root-mean-squared error of predictions generated by the random forests model to be 4.46 kg, which compares favorably to a value of 7.42 kg for a linear regression of body mass and footprint length (Supplementary Figure 1). Body mass estimates from fossil hominin trackways were therefore generated using the random forests model, with the average print dimensions for particular trackways serving as the input variables. These predictions were compared to the known body masses of the modern human experimental sample and predicted body masses from East African hominin skeletal fossils that are roughly contemporaneous with the Ileret trackways (Fig. 2B, Supplementary Table 3).

### Morphological comparisons of hominin footprints

Photogrammetric 3D models of fossil hominin footprints were quantitatively compared to an experimental data set consisting of 490 footprints created by 41 habitually barefoot Daasanach individuals (local to the Ileret area) in the same type of substrate in which the Ileret fossil tracks are preserved^24^. These data were collected as part of a previously published study^24^ and informed consent was obtained from all subjects, in accordance with a protocol approved by the Institutional Review Board of The George Washington University.

Within each trial, substrate hydration and compaction were varied, and each individual produced a set of footprints that varied in depth. We cannot know the exact mechanical properties of the substrate when the fossil tracks were produced. However, we made the experimental prints as comparable as possible by using the same sediment in which fossil footprints were formed. Further, by sampling each individual’s footprints across a variety of hydrated and compaction conditions we could incorporate substrate-driven variations into our analyses. Substrates were controlled in such a way that the range of depths of experimental prints would provide a robust sampling of the depths observed across the fossil tracks^24^.

For all of the fossil hominin and modern human tracks, photogrammetric models were oriented such that a best-fit plane through the undisturbed surface surrounding each print represented the X-Y plane (using Geomagic Studio 14; 3D Systems Inc., Rock Hill, SC). Depths were then measured at each of 14 functionally-relevant locations across each footprint that could be confidently identified (repeatability tests have shown an average error for replicate measurements of 0.005 cm), specifically the depressions beneath the medial and lateral heel, medial and lateral midfoot, all five metatarsal heads and each of the five toes.

A resampling analysis was then used to determine the overall similarity of the morphologies of the Ileret and Laetoli hominin footprints to those of modern humans. In this analysis, a separate iterative procedure was followed for each distinct fossil trackway. First, we selected from the modern human sample a subset of footprints that were created by the same gait type (walking or running) as predicted for a given fossil trackway. These predictions were generated using machine learning algorithms built using the modern human experimental data set (see Supplementary Note 4). Within the appropriate comparative data, we randomly sampled a set of footprints from an individual human subject equivalent in size (number of tracks) to the fossil trackway in question. The average topography from the randomly sampled set of footprints (average of each regional depth measurement) was computed, and the Mahalanobis distance was calculated between that morphology and the shape of the average footprint topography from the rest of the human subjects. This sampling protocol was repeated 10,000 times to generate a distribution of Mahalanobis distances that random human samples, equivalent in size (number of prints) to a given fossil trackway, fell from the average out-of-sample human footprint. The average topography of the fossil trackway was then determined, and its Mahalanobis distance from the overall average human footprint was calculated along with the probability of sampling a distance at least that large from within the resampled human data set. This provided probabilistic measures of the morphological similarity between each fossil trackway and the tracks of modern habitually barefoot people created in the same type of substrate.

### Attribution of predicted sex to Ileret hominin tracks

Attributions of predicted sex were generated using the mean method^30^. We calculated the average of all body mass predictions for the entire Ileret footprint assemblage, excluding from that calculation one extreme outlier that likely represents a child. All trackways with estimated body masses larger than the mean were predicted to be male, while those smaller than the mean were predicted to be female. This is likely to produce a conservative underestimate of the true number of males present, given evidence for considerably higher sexual dimorphism in *H. erectus* compared to modern humans^11^,^22^,^31^,^32^,^33^.

### Code availability

All analyses were performed using custom scripts written in the R programming language and environment^39^. Those custom scripts also utilized certain functions available from the ‘caret’^40^, ‘dplyr’^41^, ‘ggplot2’^42^, and ‘randomForest’^43^ packages. Computer code can be obtained through written request of the corresponding author (K.G.H.).

## Additional Information

**How to cite this article**: Hatala, K. G. *et al.* Footprints reveal direct evidence of group behavior and locomotion in *Homo erectus. Sci. Rep.* **6**, 28766; doi: 10.1038/srep28766 (2016).

## Supplementary Material

Supplementary Information

## Figures and Tables

**Figure 1:**
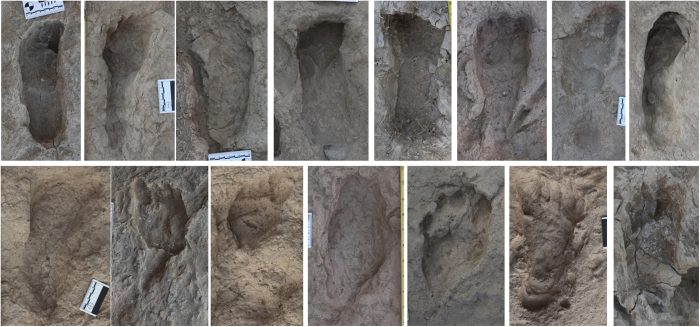
1.5 Ma hominin tracks from Ileret, Kenya. Representative images of hominin tracks uncovered in the Ileret area between 2007 and 2014. These tracks come from five different sites within about 1.5 km of each other. Some tracks show deterioration and overprinting, while many preserve fine detail, indicating that they were rapidly hardened and covered with sediment. No two sites represent the same continuous surface, as all come from different stratigraphic levels within the Ileret tuff complex. The total sample includes 97 hominin tracks produced by at least 20 different individuals.
