# Supplementary material for: Footprints reveal direct evidence of group behavior and locomotion in Homo erectus
Source: Sci Rep. 2016 Jul 12;6:28766. doi: 10.1038/srep28766 (PMC4941528; doi:10.1038/srep28766)
Supplement: Supplementary Information [file srep28766-s1.pdf]

Supplementary Materials for

Footprints reveal direct evidence of group behavior and locomotion in *Homo erectus*

Kevin G. Hatala *, Neil T. Roach, Kelly R. Ostrofsky, Roshna E. Wunderlich,
Heather L. Dingwall, Brian A. Villmoare, David J. Green,
John W. K. Harris, David R. Braun, Brian G. Richmond

* Correspondence to: kevin.g.hatala@gmail.com

This supplementary file includes:

Supplementary Notes 1-4
Supplementary Figure 1
Supplementary Tables 1-4
Supplementary References

Supplementary Note 1. *Attribution of Ileret hominin footprints to* Homo erectus – Based on their large sizes, the Ileret footprints were most likely created by *Homo erectus* individuals. Body size estimates derived from track dimensions suggest that the majority of the Ileret footprints, including at least one set of tracks at each unique site, were created by individuals with body masses that exceeded the largest skeletally-based estimates for either *Paranthropus boisei* or *Homo habilis*[1]. Some of the Ileret tracks imply smaller body masses that fall within the range of overlap in skeletally-based estimates for *H. erectus*, *P. boisei* and *Homo habilis*, raising the question of whether multiple taxa may have been present at some sites. Although this is possible, it would require those taxa to have nearly identical feet, a remarkably similar gait, and a level of sympatry and habitat overlap that included tolerance of multiple species traversing the same *c.*1-20 m^2 areas within minutes to hours of each other. There are multiple lines of evidence that would contradict this scenario. First, these three taxa have postcranial anatomies that are different enough to potentially influence differences in bipedal biomechanics[2-5]. Second, isotopic studies[6] and habitat reconstructions[7] suggest that *Homo* and *Paranthropus* consumed different diets and inhabited different environments. Isotopic evidence appears consistent with a pattern of niche partitioning that has been observed in modern populations of sympatric gorillas and chimpanzees[8,9]. Finally, modern sympatric gorillas and chimpanzees may feed in close proximity to each other during times when shared food resources are scarce but the frequency and nature of such interactions are largely unknown[10]. We cannot rule out the possibility that some of the Ileret footprints may have been produced by a taxon (or taxa) other than *H. erectus*. However, since the data are most evidently consistent with an attribution to *H. erectus*, and there is no clear and direct evidence from which to support any alternative attributions for any of the tracks, it is most parsimonious at this time to attribute them all to *H. erectus*.

Supplementary Note 2. *Potential earlier evidence of human-like foot function* – Recently discovered fossils attributed to *H. naledi* show an overall foot morphology that is remarkably human-like[11]. While these fossils are intriguing, uncertainties surrounding their geological age[12] and their taxonomic relationship to *H. erectus*[11] at this time preclude any tests of the hypothesis that they could represent evidence of human-like foot function in a pre-*H. erectus* hominin taxon.

Supplementary Note 3. *Morphological analysis of Laetoli hominin tracks* – Our results concerning the Laetoli tracks, while not the focus of this study, differ from the results of some other quantitative analyses[13-15]. The most likely explanation for our different findings is that, with one exception[15], past studies have used analytical approaches that differ from our direct statistical comparisons of overall footprint topographies. The one study that has used a similar approach[16] concluded that the shapes of the Laetoli tracks were consistent with a human-like gait. However, that study's analyses found numerous statistically significant differences between the morphologies of the Laetoli tracks and habitually shod human tracks, in the regions of the heel, midfoot, and toes[15 (Fig. 2)]. The authors argued that those significant differences were not meaningful or relevant, based on anecdotal reasoning for how and why they believe human tracks are probably more variable than those in their comparative sample.

Here, we find highly statistically significant differences (p=0 in the context of our resampling analysis) between the morphologies of the Laetoli tracks and a much larger sample of human tracks (about 5 times as large as the modern human sample size of the above-mentioned study[15]), which represent an arguably 'more relevant' sample of tracks produced by habitually barefoot people. Because tracks directly represent the external movements of the foot through a

deformable substrate, there is no reason to believe that these data and results are not strongly indicative of biomechanical differences between the gaits of the Laetoli hominins and modern humans. While the Laetoli tracks may not represent a bipedal gait that characterized every hominin taxon prior to *H. erectus*, and more human-like gaits could have existed prior to 1.5 Ma, the Ileret footprints currently offer what is currently the oldest *direct* evidence of human-like gait biomechanics.

Supplementary Note 4. *Predicting gait type from footprint trackways* – Traditional analyses of fossil hominin trackways have quantified linear relationships (via regression) between stride length and some measure of relative traveling speed using experimentally-derived data, and then used that relationship to predict traveling speeds and subsequently infer gait types (walking or running)[14,16-18]. Here, alternative methods were used that were found to more accurately classify footprint trackways as representative of walking or running gaits. A random forests model was trained to predict whether a footprint was produced during walking or running, using a footprint's internal topography (the 14 depth measurements) and the relative stride length of consecutive footfalls. This method, when tested on modern human data held out of the sample used to train the model, achieved a classification accuracy rate of 97.9%. We conducted a similar test in which we trained a linear regression model, using the relationship between relative stride length (stride length/footprint length) and dimensionless speed (Froude number), and used that model to predict Froude numbers for a set of human data held out of the training sample. From those predictions, we classified trackways with predicted Froude numbers above 0.5 as running trackways and those with predicted Froude numbers below 0.5 as walking trackways. This method yielded an out-of-sample classification accuracy rate of only 81.7%.

We also trained a random forests model to predict gait type from only the internal topography of a footprint, which was applied in cases of isolated fossil tracks (where stride length could not be measured). This model had an out-of-sample classification accuracy rate of 65.7% so while considerably better than a random guess, predictions generated from this model had comparatively low confidence. As a result, for isolated fossil hominin tracks, two sets of morphological comparisons were made, one to the experimental sample of human walking tracks and the other to the sample of human running tracks. In all of these cases, the comparisons of the fossil tracks to human walking or running prints did not yield results that differed in statistical significance.

Supplementary Figure 1 | Comparison of predictive accuracy of (A) random forests and (B) linear regression models used to predict body mass from footprint linear dimensions. In (A), predictions of body mass were generated through a random forests model that used as input variables two measures of footprint length (heel-to-hallux and heel-to-second digit), two measures of footprint width (forefoot breadth and heel breadth), and average footprint depth. In (B), predictions of body mass were generated from a linear regression model to predict body mass from a single measure of footprint length (heel-to-hallux). In both plots, the x-axis represents the predicted body mass from the given model, and the y-axis represents the observed body mass. Lines of identity (intercept = 0, slope = 1) are also plotted as visual aids. The observations plotted here were held out of the process of model creation, so they do not represent repeated data points from those used to fit the predictive models. The held-out test data set also differed for the two models, as data were randomly partitioned prior to fitting each model. Both of these measures were taken to avoid the possibility that a given model could be 'overfit' to a particular data set. Out-of-sample root mean squared error of predictions (evaluated on the held-out test data set) was 4.44 kg for the random forests model and 7.86 kg for the standard linear regression model.

A) B)

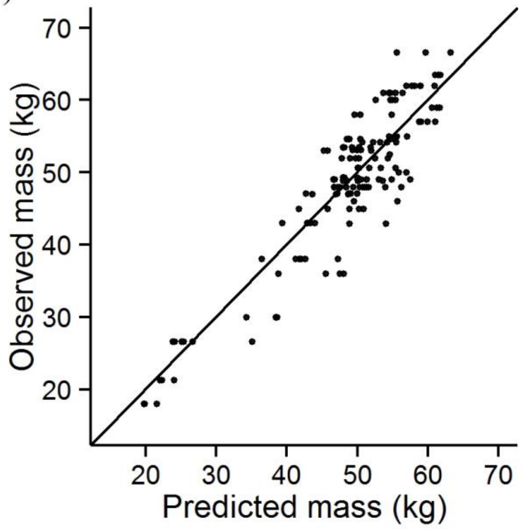

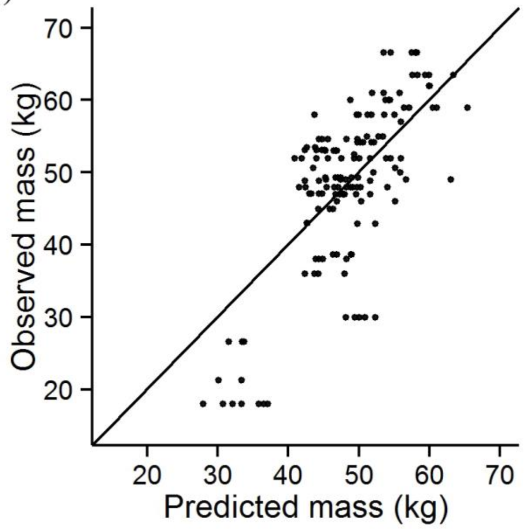

Supplementary Table 1 | Linear dimensions of Ileret, Laetoli, and modern habitually barefoot human footprints. Print dimensions were first averaged within each trackway, and between-trackway summary statistics (mean and standard deviation [SD]) are presented below. Sample sizes are presented below the means; for the fossil samples these sizes represent the number of distinct trackways while for the modern human sample the size represents the number of experimental subjects. Sample sizes for Ileret footprints differ because some prints are preserved in ways that allow measurement of footprint breadth but preclude confident measurement of footprint length.

Sample	Footprint length (cm)		Footprint breadth (cm)	
	Mean	SD	Mean	SD
Ileret	25.3<br>(n=28)	2.2	9.6<br>(n=36)	1.4
Laetoli	19.1<br>(n=1)	-	8.7<br>(n=1)	-
Modern human (Daasanach)	25.4<br>(n=41)	2.1	9.7<br>(n=41)	0.9

Supplementary Table 2 | Predicted body masses and sex attributions, derived from fossil hominin footprint dimensions. For each fossil trackway, predictions of body mass were generated using a machine learning model built on modern human data relating external footprint dimensions to body mass. Predictions of sex were determined using the mean method[19] (see also Methods). The root mean squared error of the random forests model, determined on test data that were not used in model creation, can be thought of as an estimate of 'error' that surrounds each prediction in this table. That value of root mean squared error is 4.46 kg. Some of these predictions differ from those presented in earlier publications[16] due to the discoveries of additional prints within certain trackways, and due to the use of different quantitative methods for deriving body mass predictions.

Sample	Site ID	Trackway ID	Body mass (kg)	Predicted sex
Ileret	FwJj14E Upper Footprint Layer	FUT1A	50.3	F
Ileret	FwJj14E Upper Footprint Layer	FUT1B	53.2	M
Ileret	FwJj14E Upper Footprint Layer	FUT2	53.4	M
Ileret	FwJj14E Upper Footprint Layer	FUT3	46.4	F
Ileret	FwJj14E Upper Footprint Layer	FU-C	55.7	M
Ileret	FwJj14E Upper Footprint Layer	FU-E	50.2	F
Ileret	FwJj14E Upper Footprint Layer	FU-J	51.1	M
Ileret	FwJj14E Upper Footprint Layer	FU-N	40.6	F
Ileret	FwJj14E Upper Footprint Layer	FU-O	50.8	F
Ileret	FwJj14E Upper Footprint Layer	FU-P	52.7	M
Ileret	FwJj14E Upper Footprint Layer	FU-T	52.4	M
Ileret	FwJj14E Upper Footprint Layer	FU-W	50.3	F
Ileret	FwJj14E Upper Footprint Layer	FU-X	26.5	F? (juvenile?)
Ileret	FwJj14E Upper Footprint Layer	FU-AA	58.8	M
Ileret	FwJj14E Upper Footprint Layer	FU-AB	40.4	F
Ileret	FwJj14E Upper Footprint Layer	FU-AC	47.1	F
Ileret	FwJj14E Upper Footprint Layer	FU-AD	51.1	M
Ileret	FwJj14E Lower Footprint Layer	FLT1	35.3	F
Ileret	ET-2013-1A-FE3	FE3-H11	49.4	F
Ileret	ET-2013-1A-FE3	FE3-H13	63.4	M
Ileret	ET-2013-1A-FE3	FE3-H17	60.0	M
Ileret	ET-2013-1A-FE3	FE3-H6	53.8	M
Ileret	ET-2014-3-FE8	FE8-H1	56.2	M
Laetoli	Site G	G1	23.6	-

Supplementary Table 3 | Skeletal fossil body mass estimates. Body mass estimates for published fossil skeletal sample[1] referred to in Fig. 2B. Given the geographic location of the Ileret footprints, this comparative sample of skeletally based mass estimates presented in Fig. 2B was limited to East African specimens, and it only included specimens with confident, or at least not frequently debated, taxonomic attributions as described by Grabowski and colleagues[1]. Here, for the purpose of additional comparisons, we also include additional fossils of geographic and temporal relevance but with uncertain taxonomic attributions that were not included in the calculations of Fig. 2B. These more questionably attributed specimens are indicated by asterisks (*) next to their specimen IDs.

Fossil specimen ID	Assigned taxon	Estimated body mass (kg)
KNM-ER 1808	*H. erectus*	38.5[1]
KNM-ER 3228	*H. erectus*	50.0[1]
KNM-WT 15000	*H. erectus*	53.3[1]
OH 28	*H. erectus*	62.2[1]
KNM-ER 3735	*H. habilis*	38.4[1]
OH 62	*H. habilis*	27.3[1]
OH 80-12	*P. boisei*	46.4[1]
BSN49/P27*	*H. erectus (?)*	29.4[1]
Dmanisi (D4167, D3901)*	*H. erectus (?)*	40.7[1]
Dmanisi (D3160)*	*H. erectus (?)*	35.6[1]
KNM-ER 736*	*H. erectus (?)*	65.5[1]
KNM-ER 737*	*H. erectus (?)*	64.1[1]
KNM-ER 803A*	*H. erectus (?)*	54.8[1]
KNM-ER 5428*	*H. erectus (?)*	93.4[20]
KNM-ER 1464*	*P. boisei (?)*	49.6[21]
KNM-ER 164*	*Homo* sp.	51.7[20]
KNM-ER 741*	*Homo* sp.	47.6[20]
KNM-ER 813A*	*Homo* sp.	51.8[21]
KNM-ER 1472*	*Homo* sp.	45.4[1]
KNM-ER 1475*	*Homo* sp.	42.7[1]
KNM-ER 1476A*	*Homo* sp.	32.9[21]
KNM-ER 1481*	*Homo* sp.	40.9[1]
KNM-ER 1810*	*Homo* sp.	40.5[1]
KNM-ER 3728*	*Homo* sp.	45.0[22]
KNM-ER 5880A*	*Homo* sp.	45.1[1]
KNM-ER 5881*	*Homo* sp.	35.5[1]
OH 34*	*Homo* sp.	28.3[1]
Gombore IB-7594 (MK3)*	Hominini gen. et sp. indet.	88.2[23]
KNM-ER 1471*	Hominini gen. et sp. indet.	37.2[1]
OH 35*	Hominini gen. et sp. indet.	35.5[1]

Supplementary Table 4 | Results of quantitative comparisons of Ileret and Laetoli track morphologies to those of habitually barefoot modern humans. The rightmost column represents the probability of sampling a set of human footprints with similar morphologies to the given fossil trackway. Only the best-preserved fossil hominin tracks were analyzed, in which morphological regions could be identified with confidence and where there was no evidence of taphonomic damage to the track. While the inclusion/exclusion of Ileret tracks was determined from the authors' first-hand documentation of their excavation, the well-preserved Laetoli sample was selected based on published accounts of the excavation[24,25].

Sample	Trackway ID	Tracks analyzed	Site ID	Probability of sampling similar human tracks
Ileret	FLT1	FLT1-2	FwJj14E Lower Footprint Layer	0.385
Ileret	FUT1A	FUT1-6, FUT1-12, FUT1-16	FwJj14E Upper Footprint Layer	0.091
Ileret	FU-O	FU-A, FU-M	FwJj14E Upper Footprint Layer	0.005
Ileret	FU-E	FU-H	FwJj14E Upper Footprint Layer	0.243
Ileret	FU-X	FU-Y	FwJj14E Upper Footprint Layer	0.009
Ileret	FU-AA	FU-AA	FwJj14E Upper Footprint Layer	0.198
Ileret	FU-AD	FU-AD	FwJj14E Upper Footprint Layer	0.074
Ileret	FE1-H1	FE1-H1	ET-2013-1A-FE1	0.167
Laetoli	G1	G1-25, G1-27, G1-33, G1-34, G1-35	Site G	0

Supplementary References

1 Grabowski, M. W., Hatala, K. G., Jungers, W. L. & Richmond, B. G. Body mass estimates of hominin fossils and the evolution of human body size. *J. Hum. Evol.* **85**, 75-93 (2015).

2 Johanson, D. C. *et al.* New partial skeleton of *Homo habilis* from Olduvai Gorge, Tanzania. *Nature* **327**, 205-209 (1987).

3 Wood, B. & Collard M. The human genus. *Science* **284**, 65-71 (1999).

4 Richmond, B.G., Harris, J.W.K., Mbua, E., Braun, D.R., Bamford, M., Bobe, R., Green, D.J., Griffin, N.L., McCoy, J.T., Merritt, S., Pante, M., Pobiner, B., Carter-Menn, H., Chirchir, H., Kiura, P. & Kibunjia, M. Divergence in hominin upper limb anatomy in the early Pleistocene. *Am. J. Phys. Anthropol.* 138, 221 (2009).

5 Domínguez- Rodrigo, M., Pickering, T.R., Baquedano, E., Mabulla, A., Mark, D.F., Musiba, C.M., Bunn, H.T., Uribelarrea, D., Smith, V., Diez-Martin, F., Pérez-González, A., Sánchez, P., Santonja, M., Barboni, D., Gidna, A., Ashley, G., Yravedra, J., Heaton, J.L. & Arriaza, M.C. First partial skeleton of a 1.34-Million-Year-Old *Paranthropus boisei* from Bed II, Olduvai Gorge, Tanzania. *PLoS One* 8, e80347 (2013).

6 Cerling, T.E., Mbua, E., Kirera, F.M., Manthi, F.K., Grine, F.E., Leakey, M.G., Sponheimer, M. & Uno, K.T. Diet of *Paranthropus boisei* in the early Pleistocene of East Africa. *Proc. Natl. Acad. Sci. U.S.A.* 108, 9337-9341 (2011).

7 Reed, K.E. Early hominid evolution and ecological change through the African Plio-Pleistocene. *J. Hum. Evol.* 32, 289-322 (1997).

8 Macho, G.A. & Lee-Thorp, J.A. Niche partitioning in sympatric *Gorilla* and *Pan* from Cameroon: Implications for life history strategies and for reconstructing the evolution of hominin life history. *PLOS One* **9**, e102794 (2014).

9 Oelze, V.M., Head, J.S., Robbins, M.M., Richards, M. & Boesch, C. Niche differentiation and dietary seasonality among sympatric gorillas and chimpanzees in Loango National Park (Gabon) revealed by stable isotope analysis. *J. Hum. Evol.* **66**, 95-106 (2014).

10 Stanford, C.B. & Nkurunungi, J.B. Behavioral ecology of sympatric chimpanzees and gorillas in Bwindi Impenetrable National Park, Uganda: Diet. *Int. J. Primatol.* **24**, 901-918 (2003).

11 L. R. Berger *et al.*, *Homo naledi*, a new species of the genus *Homo* from the Dinaledi Chamber, South Africa. *eLife* **4**, e09560 (2015).

12 P. H. G. M. Dirks *et al.*, Geological and taphonomic context of the new hominin species *Homo naledi* from the Dinaledi Chamber, South Africa. *eLife* **4**, e09561 (2015).

13 Tuttle, R. H. in *Laetoli: a Pliocene Site in Northern Tanzania* (eds. Leakey, M.D. & Harris, J. M.) 503-520 (Clarendon Press, Oxford, 1987).

14 D. A. Raichlen, H. Pontzer, M. D. Sockol, The Laetoli footprints and early hominin locomotor kinematics. *J. Hum. Evol.* **54**, 112 (2008).

15 Crompton, R. H. *et al.*, Human-like external function of the foot, and fully upright gait, confirmed in the 3.66 million year old Laetoli hominin footprints by topographic statistics, experimental footprint-formation and computer simulation. *J. R. Soc. Interface* **9**, 707-719 (2012).

16 Dingwall, H. L., Hatala, K. G., Wunderlich, R. E. & Richmond, B. G. Hominin stature, body mass, and walking speed estimates based on 1.5 million-year-old fossil footprints at Ileret, Kenya. *J. Hum. Evol.* **64**, 556-568 (2013).

17 J. Charteris, J. C. Wall, J. W. Nottrodt, Functional reconstruction of gait from the Pliocene hominid footprints at Laetoli, northern Tanzania. *Nature* **290**, 496 (1981).

18 R. M. Alexander, Stride length and speed for adults, children, and fossil hominids. *Am. J. Phys. Anthropol.* **63**, 23 (1984).
19 Plavcan, J. M. Comparison of four simple methods for estimating sexual dimorphism in fossils. *Am. J. Phys. Anthropol.* **94**, 465-476 (1994).
20 Boyle, E.K. & DeSilva, J.M. A large *Homo erectus* talus from Koobi Fora, Kenya (KNM-ER 5428), and Pleistocene hominin talar evolution. *PaleoAnthropology* **2015**, 1-13 (2015).
21 Pablos, A., Pantoja-Pérez, A., Martínez, I., Lorenzo, C. & Arsuaga, J. L. Metric and morphological analysis of the foot in the Middle Pleistocene sample of Sima de los Huesos (Sierra de Atapuerca, Burgos, Spain). *Quatern. Int.* **In press**, 1-11 (2015).
22 Anton, S.C. Natural history of *Homo erectus*. *Yearb. Phys. Anthropol.* **46**, 126-170 (2003).
23 Di Vincenzo, F., *et al*. The massive fossil humerus from the Oldowan horizon of Gombore I, Melka Kunture (Ethiopia, >1.39 Ma). *Quatern. Sci. Rev.* **122**, 207-221 (2015).
24 M. D. Leakey, in *Laetoli: a Pliocene site in northern Tanzania,* M. D. Leakey, J. M. Harris, Eds. (Clarendon Press, Oxford, 1987), pp. 490-496.
25 T. D. White, G. Suwa, Hominid footprints at Laetoli: facts and interpretations. *Am. J. Phys. Anthropol.* **72**, 485 (1987).
